# Microbial metabolism of isoprene: a much-neglected climate-active gas

**DOI:** 10.1099/mic.0.000931

**Published:** 2020-05-22

**Authors:** J. Colin Murrell, Terry J. McGenity, Andrew T. Crombie

**Affiliations:** ^1^​ School of Environmental Sciences, University of East Anglia, Norwich, NR4 7TJ, UK; ^2^​ School of Life Sciences, University of Essex, Wivenhoe Park, Colchester, CO4 3SQ, UK; ^3^​ School of Biological Sciences, University of East Anglia, Norwich, NR4 7TJ, UK

**Keywords:** Isoprene, microbial ecology, BVOC, atmospheric trace gas, stable isotope probing, biogeochemical cycling, *Rhodococcus*, *Variovorax*, soluble diiron centre monooxygenase

## Abstract

The climate-active gas isoprene is the major volatile produced by a variety of trees and is released into the atmosphere in enormous quantities, on a par with global emissions of methane. While isoprene production in plants and its effect on atmospheric chemistry have received considerable attention, research into the biological isoprene sink has been neglected until recently. Here, we review current knowledge on the sources and sinks of isoprene and outline its environmental effects. Focusing on degradation by microbes, many of which are able to use isoprene as the sole source of carbon and energy, we review recent studies characterizing novel isoprene degraders isolated from soils, marine sediments and in association with plants. We describe the development and use of molecular methods to identify, quantify and genetically characterize isoprene-degrading strains in environmental samples. Finally, this review identifies research imperatives for the further study of the environmental impact, ecology, regulation and biochemistry of this interesting group of microbes.

## Isoprene production in the biosphere and effects on climate

Isoprene (2-methyl-1,3-butadiene) is the most abundantly produced biogenic volatile organic compound (BVOC) in our biosphere with atmospheric emissions of around 500 Tg(C) per year. This is roughly the same as emissions of all other BVOCs combined and equal (in terms of mass of carbon) to global emissions of methane [1, 2]. Isoprene is a volatile and highly reactive compound and has major and multiple effects on global climate. The ways in which isoprene acts as a climate-active gas are complex. It can react with hydroxyl radicals to reduce the oxidative capacity of the atmosphere, which in turn increases the residence time of methane and therefore contributes to global warming. Isoprene can also react with nitrogen oxides in the atmosphere, resulting in increased levels of ozone, another greenhouse gas that influences climate, air quality and health of the biosphere [3]. Oxidation of isoprene in the atmosphere can also cause the creation of secondary aerosols, which in turn leads to cloud formation, and thus to an increase in the Earth’s albedo (the extent to which the planet reflects solar radiation back into space), and hence global cooling. Thus, the effects of isoprene are complex and not fully understood, and depending on the composition of other chemical species in the atmosphere, isoprene can act as both a climate-warming and a climate-cooling gas [4, 5].

**Fig. 1.**
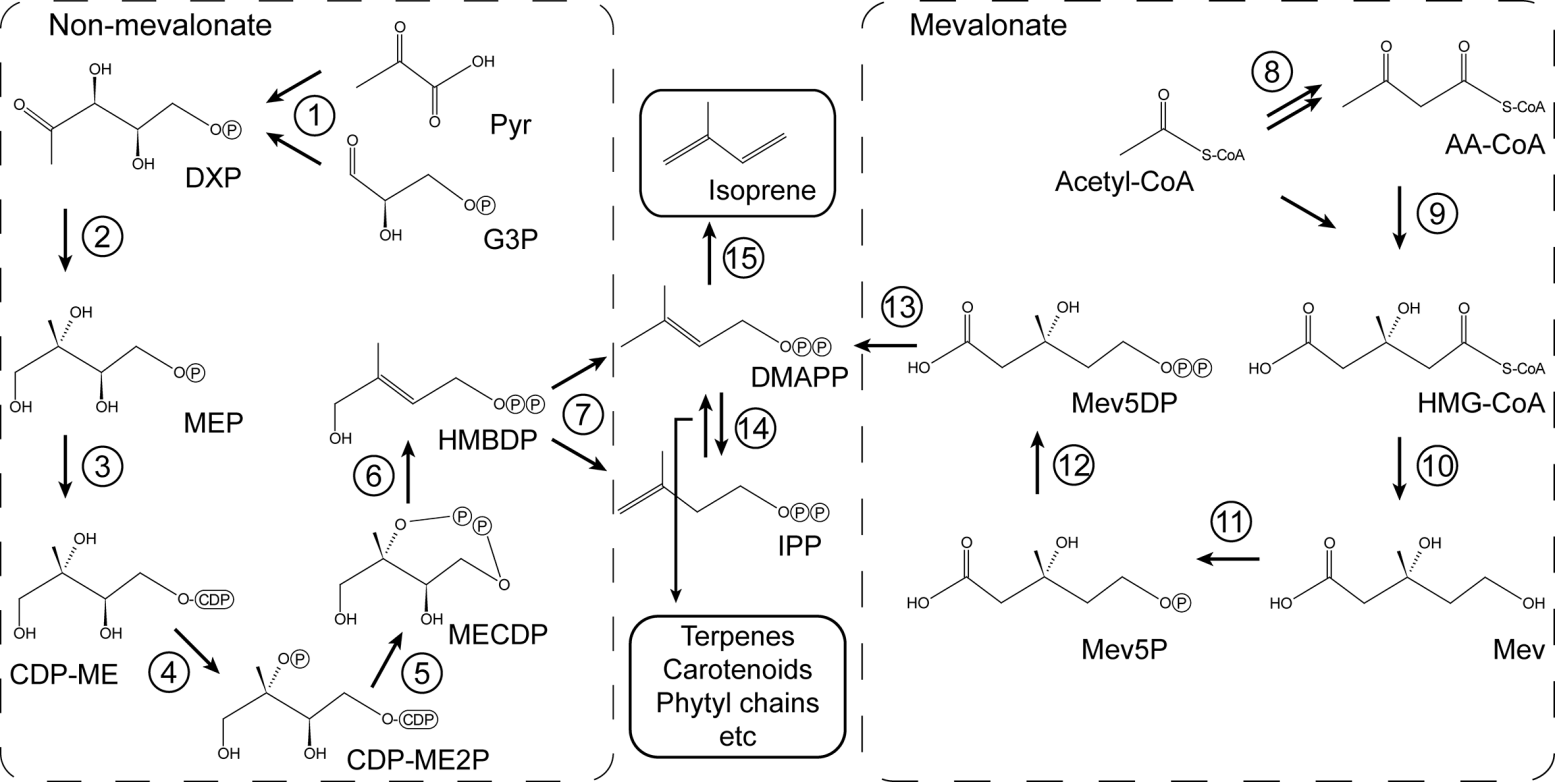
Non-mevalonate (methylerythritol 4-phosphate, MEP) and mevalonate (MVA) pathways for biosynthesis of isoprenoid precursors isopentenyl diphosphate and dimethylallyl diphosphate. Pyr, pyruvate; G3P, glyceraldehyde-3-phosphate; DXP, 1-deoxy-d-xylulose 5-phosphate; MEP, 2-*C*-methyl-d-erythritol 4-phosphate; CDP-ME, 4-(cytidine 5′-diphospho)−2-*C*-methyl-d-erythritol; CDP-ME2P, 2-phospho-4-(cytidine 5′-diphospho)−2-*C*-methyl-d-erythritol; MECDP, 2-*C*-methyl-d-erythritol 2,4-cyclodiphosphate; HMBDP, 1-hydroxy-2-methyl-2-butenyl 4-diphosphate; AA-CoA, acetoacetyl-CoA; HMG-CoA, 3-hydroxy-3-methylglutaryl-CoA; Mev, mevalonate; Mev5P, mevalonate 5-phosphate; Mev5DP, mevalonate 5-diphosphate; IPP, isopentenyl diphosphate; DMAPP, dimethylallyl diphosphate. Enzymes: 1, DXP synthase; 2, DXP reductoisomerase; 3, MEP cytidylyltransferase; 4, CDP-ME kinase; 5, MECDP synthase; 6, HMBPP synthase; 7, HMBPP reductase; 8, acetoacetyl-CoA thiolase; 9, HMG-CoA synthase; 10, HMG-CoA reductase; 11, mevalonate kinase; 12, phosphomevalonate kinase; 13, mevalonate diphosphate decarboxylase; 14, isopentenyl diphosphate isomerase; 15, isoprene synthase.

Isoprene units are key chemical building blocks for all cells, and the resulting isoprenoids in which they occur comprise a large and very diverse family of biomolecules including sterols, hopanoids, archaeal lipids, carotenoids, chlorophyll, quinones, many signalling molecules and hormones. The precursor molecules, isopentenyl diphosphate (IPP) and dimethylallyl diphosphate (DMAPP), are synthesized by either the mevalonate (MVA) pathway or the methylerythritol 4-phosphate (MEP) pathway (also known as the non-mevalonate pathway), which use as precursors acetyl-CoA, or pyruvate and glyceraldehyde 3-phosphate, respectively [6] (Fig. 1). The MVA pathway occurs in the cytosol of plants, in animals, fungi, Archaea and some bacteria. The MEP pathway operates in chloroplasts and most bacteria. DMAPP is the substrate for the key final step in the production of isoprene, catalysed by the Mg^2+^-requiring enzyme, isoprene synthase (ISPS).

Over 90 % of the isoprene produced globally comes from the conversion of DMAPP to isoprene by isoprene synthase in the chloroplasts of terrestrial plants and in particular trees and shrubs [7]. It is interesting that not all plants produce isoprene, with some high producers and some low or non-producers. There seems to be no logical pattern to isoprene production, which varies widely sometimes even in the same genus. For example, among the genus *Quercus*, American oaks produce a lot of isoprene whereas many European oaks are low isoprene producers [8]. In trees that produce large amounts of isoprene, typically 2%, although sometimes much more, of the carbon fixed by the plant can be diverted for the synthesis of isoprene [9], which cannot be stored by the plant and is lost to the atmosphere. This is a considerable loss of carbon, energy and reducing power from the plant, especially considering that it is estimated that 20 molecules of ATP and 14 molecules of NADPH are required to synthesize one molecule of isoprene [9]. There appear to be multiple roles for isoprene in plants, although there is still considerable uncertainty regarding a unifying role for this compound. Key functions might include protection against thermal and oxidative stress by altering membrane properties, or quenching reactive oxygen species, and there is increasing evidence that isoprene can affect plant growth and provides defence against herbivory by grazing insects, by acting as a signalling molecule [7, 10–12]. Recent studies also reveal that isoprene influences gene expression and the proteome and metabolome of plants [13]. The biological role for isoprene in plants is a continuing matter for debate and has been reviewed recently by Lantz *et al*. [14] and Sharkey and Monson [15]. Since trees are the major source of isoprene globally [7], there is increasing interest in the potential impact on air quality of certain crop plants [16]. For example, in temperate regions, poplar and willow are being grown as biofuel crops and one of the highest isoprene-emitting trees, oil-palm, is being grown as a crop over vast areas of the tropics. There are concerns about the impacts of these high point sources of isoprene and their effects on climate [17]. Recently, engineering of agroforest trees (hybrid poplar) to reduce leaf isoprene emissions has been demonstrated. Interestingly, in field trials, woody biomass production in these trees was not significantly reduced [18].

In addition to plants, isoprene can also be produced by some bacteria, fungi, protists, algae and animals (but not, to our knowledge, by Archaea) [7, 19–21], which together account for approximately 10 % of predicted isoprene emissions globally. The changes in human isoprene exhalation associated with diverse health conditions are being tested and in some cases exploited as a diagnostic tool, e.g. due to a connection with cholesterol biosynthesis [22]. Even external stimuli, such as a goal at a football match, can increase isoprene production in humans [23].

Of the few bacteria from the terrestrial environment that have been tested, it was shown that some Proteobacteria, Actinobacteria and Firmicutes can produce isoprene. Probably the most well-studied of these is *Bacillus subtilis,* which may produce isoprene in response to stress [24], although it is still uncertain exactly why microbes produce isoprene.

Microalgae and macroalgae are thought to be the main producers of isoprene in the marine environment [21]. Estimates of isoprene emissions from the marine environment vary considerably and range from 0.1 to 11.6 Tg(C) per year depending on the methods used for their estimation (reviewed in Shaw *et al*. [25] and McGenity *et al*. [26]). The reasons why isoprene is produced by algae in the marine environment are unclear, but again protection against high temperature, light intensity and oxidative stress may play a role [27–29].

Although highest isoprene emissions are characteristic of tropical forests, significant isoprene is also released from environmentally sensitive boreal regions which may be particularly responsive to climate change [30, 31]. The vascular and non-vascular plants in peatlands, wetlands, tundra and forests all contribute to isoprene emissions in northern latitudes [32–35], which may be strongly influenced by thawing of arctic tundra and fens [36, 37]. Other potential sources of isoprene in the environment include freshwater lakes, which have not been investigated in detail. Steinke and colleagues recently identified a substantial flux from Lake Constance (Germany, Switzerland, Austria), an oligotrophic lake that serves as a model for northern temperate deep lakes. The data showed that arctic lakes, which are often a prominent feature of boreal landscapes, may also support similar levels of isoprene emissions. The flux per unit area was similar in magnitude to that of arctic tundra vegetation, suggesting that lakes might also be a substantial source of isoprene in the rapidly warming Arctic [38].

Isoprene also has an anthropogenic source (apart from isoprene-producing crop plants), principally from vehicle exhausts which, although minor in global terms, may be important for air quality in urban areas [39–41].

While isoprene synthase from trees such as poplar has been studied in detail [42], little is known about isoprene synthases of microbes. The enzyme from *Bacillus subtilis *, although labile, was partially purified and found to have a lower divalent cation requirement and lower pH optimum than isoprene synthases from plant chloroplasts [43]. The mechanisms of isoprene production by bacteria are uncertain and are therefore a priority area for study in the future, especially since microbes have received attention recently as cell factories for the production of isoprene. Isoprene is an important commodity chemical that is industrially produced from petroleum in substantial amounts (approximately 1 million tonnes globally per year). It is used for production of polyisoprene (synthetic rubber) and has also received attention as a potential fuelstock. Approaches used to explore isoprene production in microbes have been recently reviewed by Ye *et al*. [44] and include expression of isoprene synthase genes from plants in *Escherichia coli *, *Saccharomyces* and *Synechococcus *, and enhancing carbon flux to DMAPP in microbes.

## Consumption of isoprene by microbes

It is now over 20 years since the benchmark experiments of Cleveland and Yavitt [45, 46] who demonstrated with laboratory microcosms and field-chamber methods that soils from boreal, temperate and tropical environments could consume isoprene, added at concentrations of around 400 to 500 ppbv, very rapidly down to concentrations below the limits of detection (<5 ppbv). These experiments provided the first clues as to the operation of a biological sink for isoprene in the environment (reviewed in Fall and Copley [20]). Subsequently, mesocosm experiments with temperate agroforest and tropical rainforest soil samples [47, 48] and temperate forest soils [49] have also revealed *in situ* microbial consumption of isoprene. More recently, leaves from isoprene-producing trees such as willow, poplar and oil-palm have been shown to consume isoprene in laboratory microcosm experiments [50–54] (Gibson *et al*., unpublished), indicating that the phyllosphere is also likely to be a rich source of isoprene-degrading microbes (see later).

The first reports of microbial consumption of isoprene in the marine environment were by Acuña Alvarez *et al*. [55], who determined that isoprene-degrading microbes were present in the Colne Estuary, UK, a brackish lagoon in Etang de Berre, France, and in coastal environments in Indonesia. In an elegant experiment using headspace-connected flasks they also demonstrated that isoprene produced at environmentally relevant concentrations by microalgal cultures could support growth of isoprene-degrading bacteria.

**Fig. 2.**
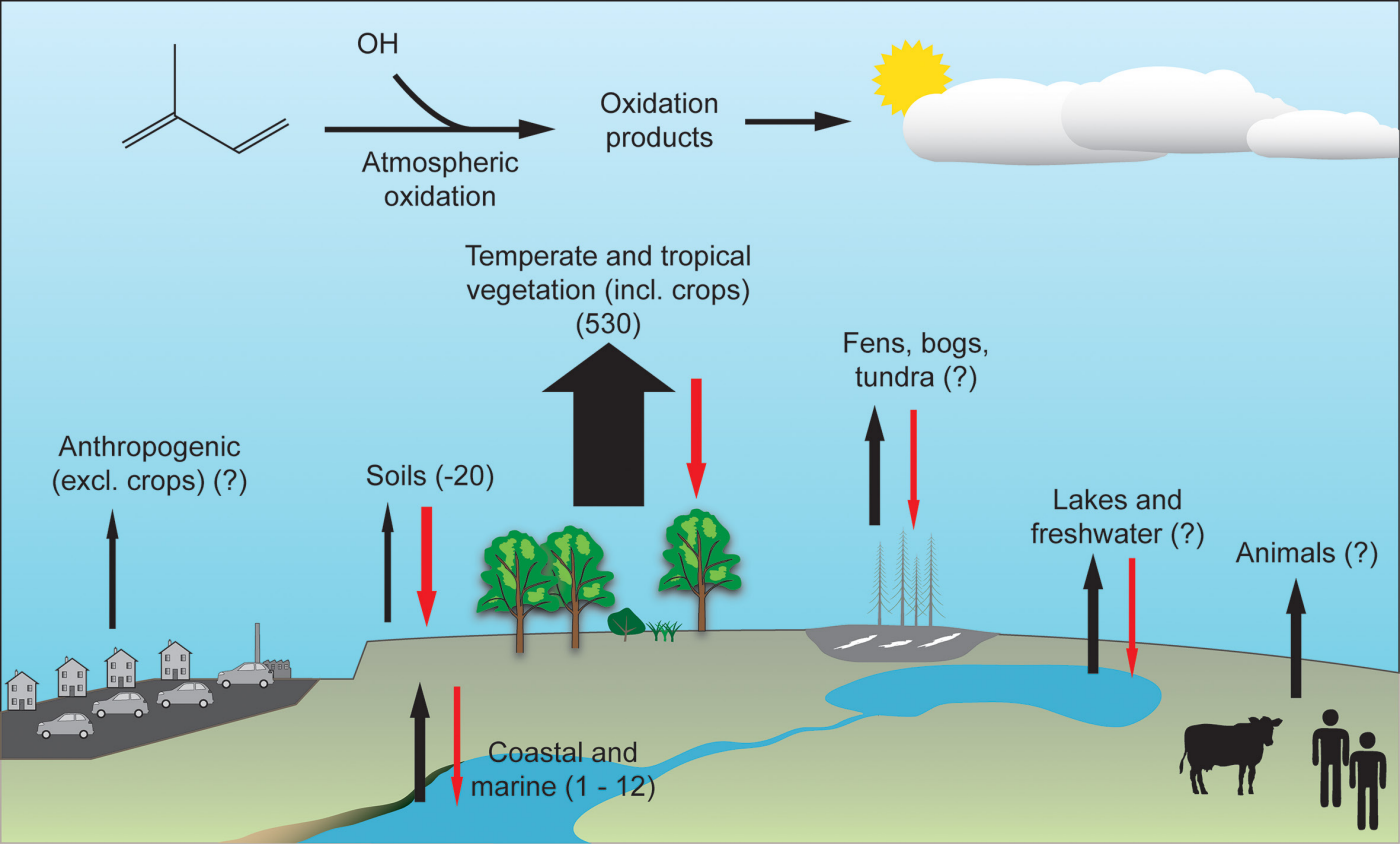
Isoprene budget. Sources are shown with black arrows and microbial sinks with red arrows. The net contribution of each component is shown in Tg(C) y^−1^, or with a question mark where data are not available [1, 25, 26, 45].

The data quantifying the biological sink for isoprene are sparse, but suggest that uptake by soils equates to the removal of 4 % of global emissions, with uncertain or unknown contributions from the marine environment and plant-associated microbes [45, 47, 49, 56, 57]. Similarly, uncertainties exist over the marine isoprene source and the global effects of climate change on isoprene emissions [5, 26]. Estimates of the current sources and sinks are shown in Fig. 2.

## Bacterial growth on isoprene

Aerobic growth of bacteria on isoprene was first described by van Ginkel and colleagues [58, 59], Ewers *et al*. [60] and Cleveland and Yavitt [45], who isolated actinobacterial strains tentatively assigned to the genera *Nocardia *, *Rhodococcus* and *Arthrobacter,* and a proteobacterial *Alcaligenes* strain, from soil enrichments with isoprene. Estuarine marine sediments have also yielded a number of both Gram-positive and Gram-negative isoprene-degrading bacteria, the most well characterized being *Gordonia* and *Mycobacterium* strains [55, 61]. Isoprene-degrading *Pseudomonas, Klebsiella* and *Alcaligenes* strains have also been isolated from rubber-contaminated soils but these have not been characterized in detail [62]. A *Methylobacterium* strain isolated from agricultural soil was able to cometabolize isoprene while using methane (unusually for a member of this genus) as the primary growth substrate [63]. However, there is also evidence that representatives of this genus can grow on isoprene as the sole source of carbon and energy [53] (Murphy *et al*., unpublished).

More recently, the phyllosphere has been shown to harbour isoprene degraders. For example, leaf enrichments from isoprene-emitting poplar trees yielded further *Rhodococcus* strains, a *Pseudonocardia* strain and a Gram-negative *Variovorax* strain [50]. Similarly, leaves of willow (also a significant isoprene-emitter) and soil from the vicinity of these trees have yielded Gram-positive isoprene-degrading strains including *Nocardioides *, *Rhodococcus* and *Gordonia *, together with new Gram-negative strains of the genera *Ramlibacter *, *Variovorax* and *Sphingopyxis* [52]. Isoprene degraders from the genera *Arthrobacter *, *Bacillus, Pseudomonas*, *Sphingobacterium *, *Sphingobium* and *Pantoea* were isolated from the leaf surface of the tropical tree species *Madhuca latifolia* (mahua) and *Tectona grandis* (teak) [54]. The presence of several of these strains was revealed by cultivation-independent approaches using DNA-stable isotope probing (DNA-SIP), which also helped to guide their isolation (see later). All the isoprene degraders described above are aerobes. Recently, there has been one report hinting that anaerobic degradation of isoprene may be an overlooked mechanism for isoprene consumption in the environment. Kronen *et al*. [64] reported that a hydrogen-consuming homoacetogenic enrichment carried out reductive metabolism of isoprene to a mixture of methylbutene isomers, thus indicating that strict anaerobes can use isoprene as an electron acceptor to support homoacetogenesis. It remains to be seen whether anaerobes can also use isoprene as the sole carbon and energy source for growth. Although soil incubations identified enrichment of some fungi (in addition to bacteria) in response to atmospherically relevant concentrations of isoprene [49], to our knowledge there have been no conclusive reports of fungi or Archaea growing on isoprene as the sole carbon and energy source. In addition, cultivation-independent methods and surveys (see later) have not revealed the definitive presence of such degraders in environmental samples examined to date. A comprehensive list of extant isoprene-degrading bacteria, and the environments from which they were isolated is presented in Table 1.

**Table 1.** Isoprene-degrading bacterial strains

Taxon/strain name	Reference	IsoA accession no.	Source*	Plant†	Country‡	Genome accession no.
**Actinobacteria**						
*** Arthrobacter ***						
Not specified	[46]		Soil		USA	
BHU_FT2	[54]		Soil	*T. grandis*	India	
BHU_FM3	[54]		Soil	*M. latifolia*	India	
*** Gordonia ***						
i37	[55, 61]	ANQ29701.1	Est		UK	ASM204308v1
OPL2	(in prep)	AZL41296.1	Leaf	*E. guineensis*	UK	
*** Leifsonia ***						
i49 (first named as i47 [45])	[55, 61]	ANQ29692.1	Est		UK	
*** Micrococcus ***						
i61b	[61]	ANQ29694.1	Est		UK	
*** Mycobacterium ***						
i61a	[61]	ANQ29693.1	Est		UK	
AT1	[61]	ANQ29700.1	Est		UK	ASM204309v1
*** Nocardia ***						
IP1 and others	[58, 59]		Soil		–	
*** Nocardioides ***						
WS12	[52]	AZL41292.1	Soil	*Salix alba*	UK	
*** Rhodococcus ***						
AD45§	[65]	KJF19164.1	FW		–	ASM94930v1
JE77	[60]				–	
i24 and i48	[55]		Est		UK	
i47	[61]	ANQ29697.1	Est		UK	
i8a2	[61]	ANQ29698.1	Est		UK	
i29a2	[61]	ANQ29699.1	Est		UK	
LB1	[72]	KXX62729.1	Leaf	*A. hippocastanum*	UK	ASM158345v1
SC4	[72]	KXF49860.1	Soil		UK	ASM155547v1
PD630	[68, 84]	EHI47089.1	Soil		Germany	ASM23433v1
ACPA1	[50, 85]	PBC57749.1	Leaf	*Populus alba*	UK	ASM230019v1
ACPA4	[50, 85]	PBC35845.1	Leaf	*Populus alba*	UK	ASM230018v1
ACS1	[50, 85]	PBC51908.1	Soil	*Salix fragilis*	UK	ASM230015v1
ACS2		AZL41284.1	Soil	*Salix fragilis*	UK	
TD1	[51]	AZL41294.1	TDS		UK	
TD2	[51]	AZL41295.1	TDS		UK	
WS1	[51]	AZL41285.1	Soil	*Salix alba*	UK	ASM379774v1
WS3	[52]	AZL41286.1	Soil	*Salix alba*	UK	ASM379708v1
WS4	[52]	AZL41287.1	Soil	*Salix alba*	UK	ASM654360v1
WS5	[51]	AZL41288.1	Soil	*Salix alba*	UK	
WS7	[52]	AZL41289.1	Soil	*Salix alba*	UK	ASM654361v1
WS8	[51]		Soil	*Salix alba*	UK	
WS10	[51]	AZL41291.1	Soil	*Salix alba*	UK	
WL1	[51]	AZL41293.1	Leaf	*Salix alba*	UK	
OPL1	[51]		Leaf	*E. guineensis*	UK	
SK2ab	(in prep)	AZL41299.1	SM		UK	
SK5	(in prep)	AZL41298.1	SM		UK	
**Firmicutes**						
*** Bacillus ***						
BHU_FM1	[54]		Soil	*M. latifolia*	India	
**Alphaproteobacteria**						
*** Loktanella ***						
i8b1	[61]	ANQ29691.1	Est		UK	
*** Methylobacterium ***						
PV1	[63]||		Soil		India	
*** Shinella ***						
i39	[55]	ANQ29696.1	Est		UK	
*** Sphingobacterium ***						
BHU_LFT1	[54]		Leaf	*T. grandis*	India	
*** Sphingobium ***						
BHU_LFT2	[54]		Leaf	*T. grandis*	India	
*** Sphingopyxis ***						
OPL5	[52]	AZL41297.1	Leaf	*E. guineensis*	UK	
*** Stappia ***						
iL2	[61]	ANQ29695.1	SW		UK	
**Betaproteobacteria**						
*** Alcaligenes ***						
JE75	[60]		–		–	
ISO 1	[62]		Soil		India	
*** Ramlibacter ***						
WS9	[52]	ROZ78065.1	Soil	*Salix*	UK	ASM379776v1
*** Variovorax ***						
WS11	[50, 52]	PSL86057.1	Soil	*Salix alba*	UK	ASM301487v1
WS13	[51]		Soil	*Salix alba*	UK	
**Gammaproteobacteria**						
*** Klebsiella ***						
ISO 2	[62]		TDS		India	
*** Pantoea ***						
BHU_LFM3	[54]		Soil	*M. latifolia*	India	
*** Pseudomonas ***						
ISO 3, ISO 4 and ISO 5	[62]		Soil		India	
BHU_FT1 and	[54]		Soil	*T. grandis*	India	
BHU_LFM1	[54]		Leaf	*M. latifolia*	India	
ML2	[86, 87]||		Soil		–	

*Source of isolate: SW, seawater; Est, estuary; FW, freshwater sediment; SM, saltmarsh sediment; TDS, tyre-dump soil.

†Plant: the source plant or the dominant plant (for plant-associated soil samples).

‡Country: origin of sample; a hyphen indicates information not available.

§Transcription of the isoprene-induced genes is also discussed in ref [58].

||The authors indicate that isoprene was co-metabolized rather than assimilated.

*T. grandis, Tectonia grandis; M. latifolia, Madhuca latifolia; E. guineensis, Elaeis guineensis; A. hippocastanum, Aesculus hippocastanum.*

## Aerobic metabolism of isoprene

**Fig. 3.**
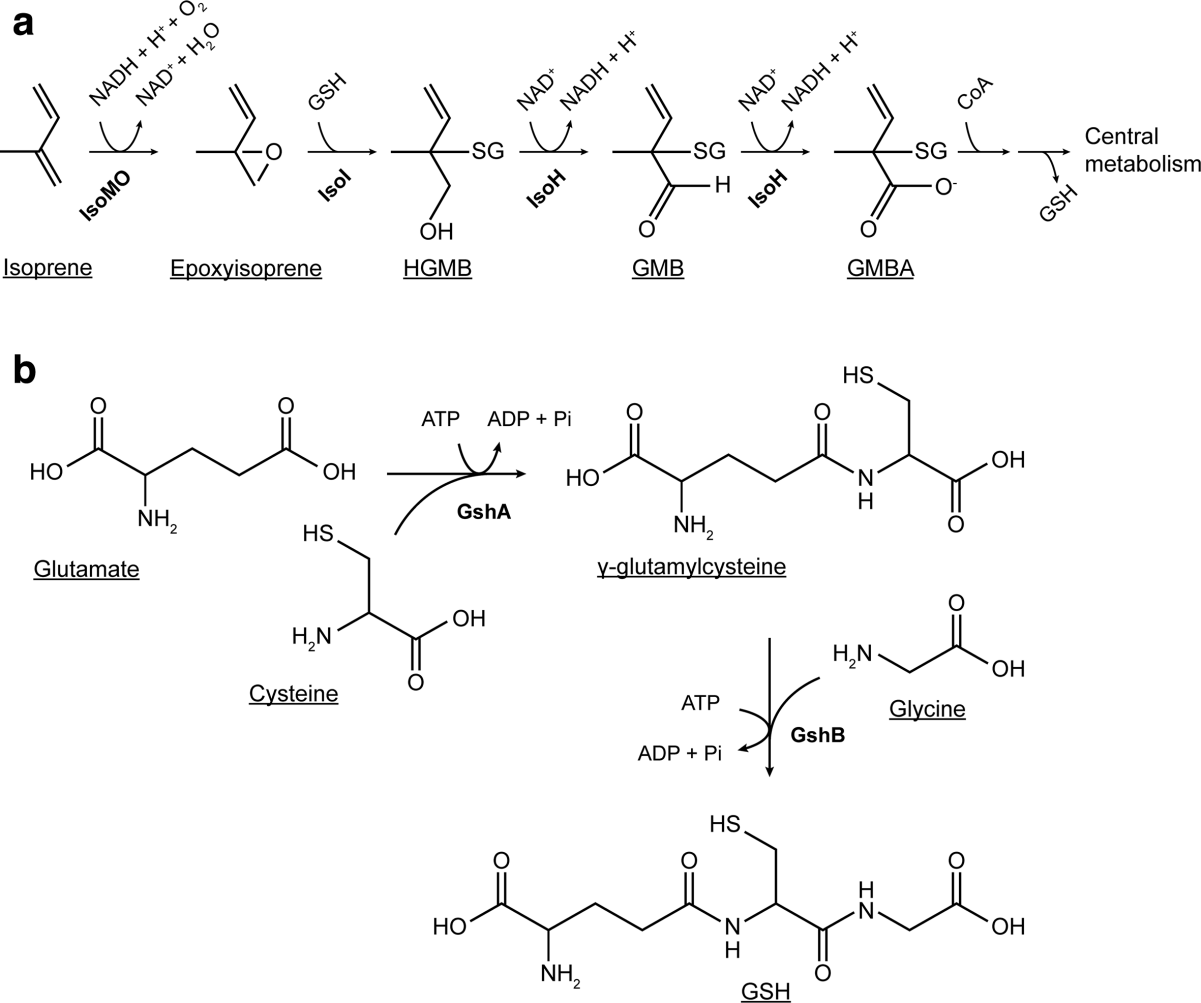
(a) Pathway of aerobic bacterial isoprene degradation and (b), of glutathione biosynthesis. HGMB, 1-hydroxy-2-glutathionyl-2-methyl-3-butene; GMB, 2-glutathionyl-2-methyl-3-butenal; GMBA, 2-glutathionyl-2-methyl-3-butenoate; G, glutathione; GSH, reduced glutathione. Enzymes: IsoMO, isoprene monooxygenase; IsoI, glutathione S-transferase; IsoH, 2-glutathionyl-2-methyl-3-butenol dehydrogenase; GshA, glutamate-cysteine ligase; GshB, glutathione synthetase.

The most well-characterized isoprene-degrading species, which has become the ‘workhorse’ in studies on the metabolism of isoprene, is *Rhodococcus* strain AD45, which was isolated from freshwater sediment and first described by Janssen and colleagues [65]. In pioneering work they proposed a putative pathway for isoprene degradation in which the initial oxidation of isoprene to 1,2-epoxyisoprene is catalysed by isoprene monooxygenase (IsoMO) and the epoxide formed is further metabolized by a glutathione transferase [65–67]. Sequencing of the 6.8 Mbp genome of *Rhodococcus* AD45 revealed that all of the genes required for isoprene metabolism are found on a 300 kbp plasmid in this *Rhodococcus* strain [68]. These genes were induced by growth on isoprene or epoxyisoprene, the latter or another subsequent oxidation product being the likely inducer. All isoprene-degrading bacteria thus far characterized contain ten core *iso* genes contained in two isoprene metabolic gene clusters *isoABCDEF* and *isoGHIJ*, which are essential for growth on isoprene. The IsoMO, catalysing the first step in the metabolism of isoprene, is a four-component enzyme of the soluble diiron centre monooxygenase (SDIMO) family [69]. The oxygenase component is encoded by *isoA*, *isoB* and *isoE* (α_2_β_2_γ_2_), while *isoC*, *isoD* and *isoF* encode a Rieske-type ferredoxin, a coupling protein and a flavoprotein NADH reductase, respectively. All four components are required for reconstitution of activity *in vitro* (Lockwood, Murrell *et al*., unpublished). Further metabolic steps are encoded by *isoGHIJ*. The expoxide produced from the initial oxidation of isoprene is likely to be toxic to the cell and so is conjugated with glutathione to form 1-hydroxy-2-glutathionyl-2-methyl-3-butene (HGMB) by a glutathione *S*-transferase (IsoI) [65] (Fig. 3). HGMB is then metabolized to 2-glutathionyl-2-methyl-3-butenoate (GMBA) by a dehydrogenase (IsoH) [66]. Further steps in the metabolism of GMBA have not been confirmed, but it is likely that the glutathione moiety is subsequently removed and β-oxidation of the intermediates of isoprene metabolism enables growth on isoprene as the sole carbon and energy source. Detoxification of epoxyisoprene by conjugating with glutathione may be a feature common to all isoprene degraders, and this mechanism is especially intriguing in Gram-positive bacteria, in which glutathione synthesis is an unusual feature [70]. In *Rhodococcus* AD45, the megaplasmid carrying *iso* genes contains duplicate copies of *isoGHIJ,* and also duplicate copies of *gshA* encoding glutamate cysteine ligase, which catalyses the first step of glutathione biosynthesis. This may be an ‘insurance policy’ to ensure that any epoxyisoprene produced is rapidly removed during growth on isoprene.

Curing of the plasmid containing *iso* genes, and also mutagenesis of *isoA*, results in loss of ability of *Rhodococcus* AD45 to grow on isoprene. Regulation of *iso* genes has been studied in detail in this isoprene degrader, in which isoprene metabolism is an inducible trait [68]. Sugars and carboxylic acids, such as glucose and succinate respectively, repress isoprene metabolism. When succinate-grown cells were subcultured into medium containing isoprene, epoxyisoprene, glucose, succinate or no substrate (as controls) and the transcriptome examined over a time-course of several hours, it was revealed that isoprene and epoxyisoprene induced very high levels of expression (~25 % of all transcripts observed) of 22 genes clustered on the megaplasmid of *Rhodococcus* AD45, including i*soABCDEF* and *isoGHIJ* [68]. Further targets for regulatory studies (which are underway in our laboratory) were revealed during this study, including three genes (*marR1*, *marR2* and *gntR*) encoding three putative transcriptional regulators and two genes predicted to encode aldehyde dehydrogenases (*aldH1* and *aldH2*) on the megaplasmid in *Rhodococcus* AD45, which are also present in the genomes of other isoprene degraders.

**Fig. 4.**
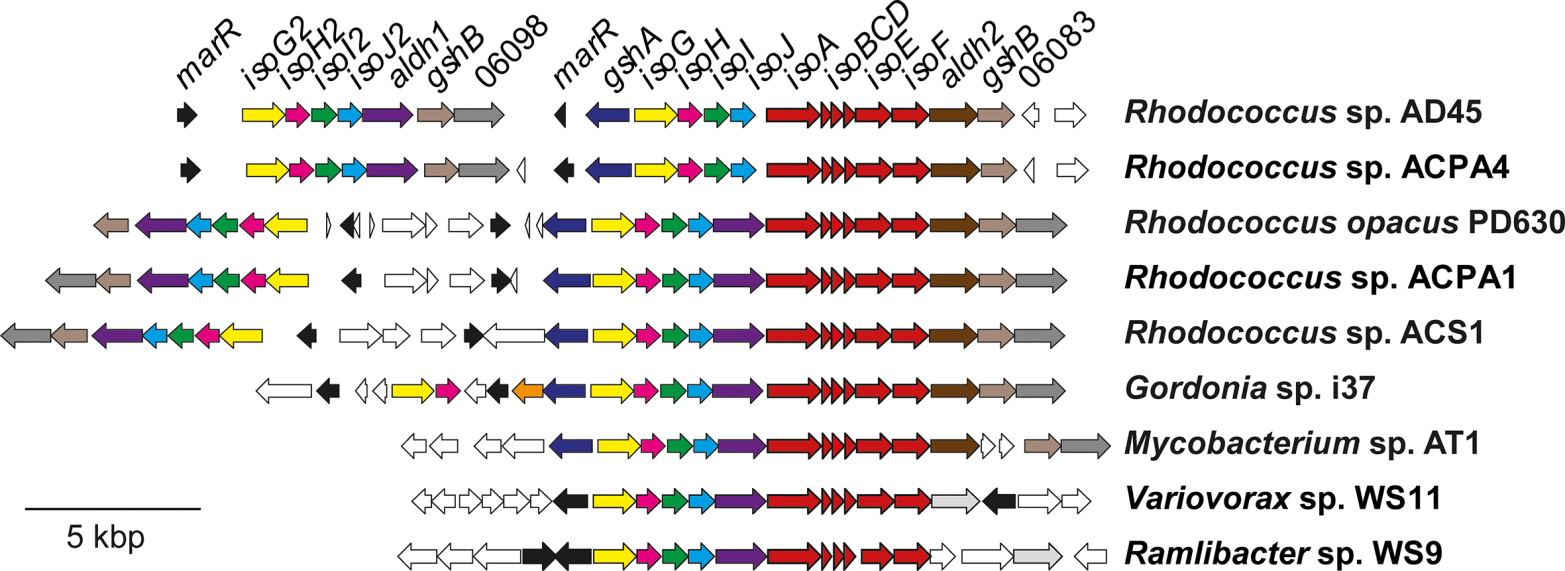
Isoprene (*iso*) gene clusters from *Rhodococcus* sp. AD45 and other representative isoprene degraders. ORFs are colour-coded according to known or putative function, with the isoprene monooxygenase shown in red. Gene identification numbers (locus tags) refer to the *R*. sp. AD45 genome sequence.

**Fig. 5.**
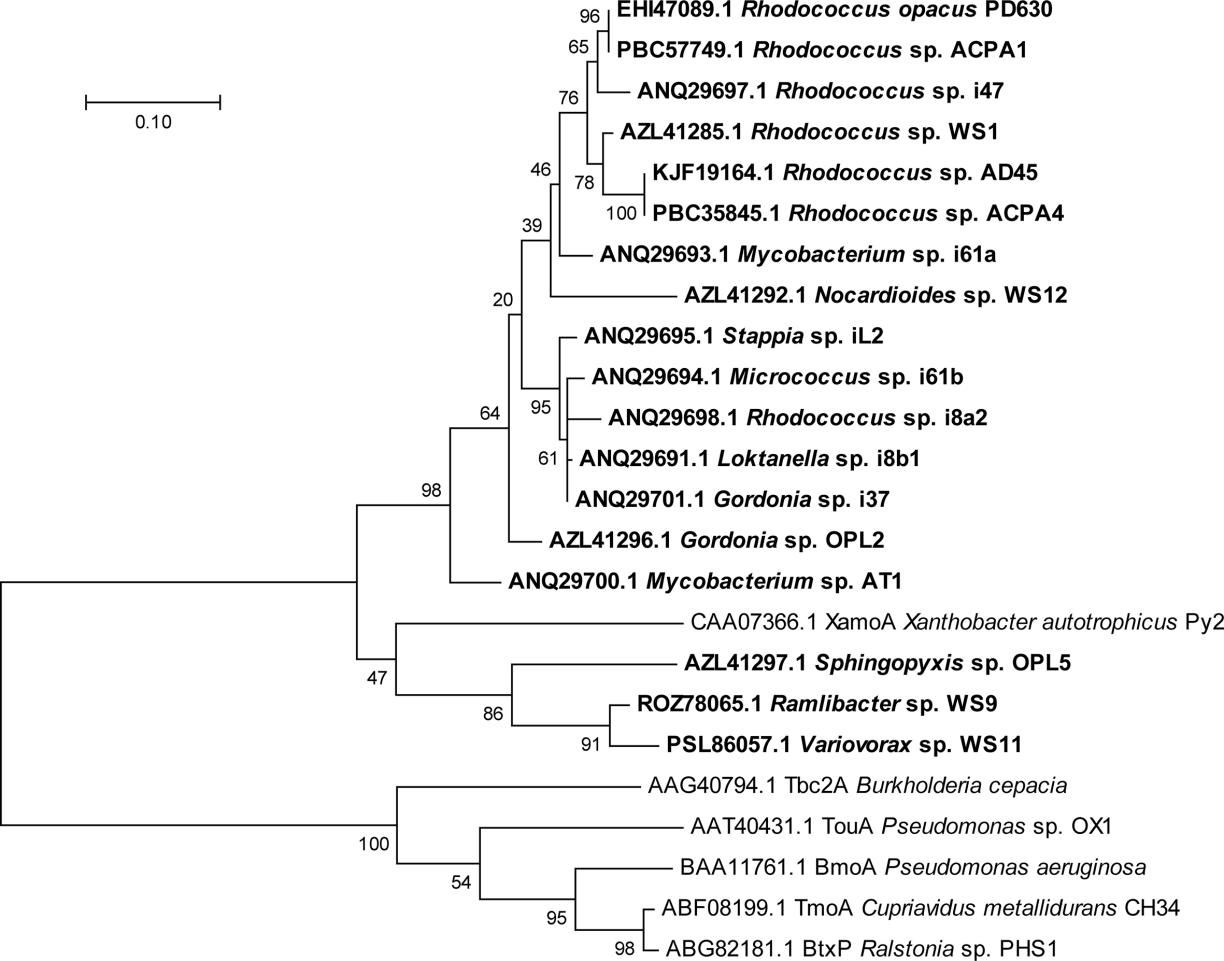
Phylogenetic tree showing the relationship of IsoA from isoprene-degrading bacteria (shown in bold type), together with homologous sequences from characterized bacteria that do not degrade isoprene, constructed using the maximum-likelihood method in mega 7 [88]. Positions containing gaps or missing data were removed, and the final alignment comprised 334 amino acids. Bootstrap values (500 replications) are shown at the nodes and the scale bar indicates substitutions per site.

The *iso* gene cluster of *Rhodococcus* AD45 together with the corresponding *iso* gene clusters from other representative isoprene degraders are shown in Fig. 4. Since IsoMO is a member of the SDIMO family of enzymes, there is a considerable degree of identity with other examples from this group, particularly with homologues of *isoA*, encoding the putative active site-containing component of IsoMO. Until recently, routine annotation of the genomes of bacteria containing SDIMO enzymes, such as toluene and alkene monooxygenase, has frequently resulted in the mis-annotation of IsoMO, typically as toluene monooxygenase. However, due to the increase in the number and diversity of cultivated isoprene degraders, and the availability of DNA sequences of *isoABCDEF* from these bacteria, it is now possible to distinguish between IsoMO and other SDIMOs, with IsoA proteins forming distinct clades within the corresponding proteins of the SDIMO family (Fig. 5). Establishment of databases for *iso* genes from extant isoprene-degrading bacteria is now enabling focussed cultivation-independent studies (see below) on isoprene degraders in the environment. For example, identification of *isoA* genes retrieved by PCR from DNA samples extracted from environmental samples, or the presence of the gene clusters *isoABCDEF* and *isoGHIJ* (encoding IsoMOs and glutathione transferases respectively) in metagenome-assembled genomes of putative isoprene degraders, is now providing robust methodology to investigate the distribution and diversity of isoprene degraders in the environment.

## Ecology of isoprene-degrading bacteria

Molecular ecology studies on aerobic methane-oxidizing bacteria (methanotrophs) have been enhanced over the years by the use of methane monooxygenase-specific gene markers such as *pmoA* and *mmoX,* encoding particulate and soluble methane monooxygenase respectively [71]. Using similar approaches we have developed *isoA* (a homologue of *mmoX*) as a functional-gene marker to detect isoprene-degrading bacteria in environmental samples. Using this marker gene, which encodes the putative active site-containing polypeptide of the oxygenase component of IsoMO, rather than relying on identifications using 16S rRNA genes alone, has been essential because, unlike methanotrophs, which are generally tightly clustered into distinct phylogenetic groups, isoprene degradation is a widespread trait across many genera (see Table 1). The *isoA* gene is highly conserved in all isoprene degraders studied to date and can be distinguished from other SDIMO homologues (Fig. 5). Alignment of IsoA sequences from extant isoprene degraders allowed the design of *isoA*-specific PCR primers that did not amplify SDIMO genes from isoprene non-degraders [72]. These *isoA* primers, when used with DNA extracted from enrichment cultures and environmental DNA isolated from various soils, sediments and leaf samples, yielded IsoA amino acid sequences which, when compared with IsoA from extant isoprene-degraders, were relatively highly conserved (>86% identity) and clearly distinct from SDIMO homologues of isoprene non-degraders. IsoA sequences retrieved from the marine environment also clustered separately from those retrieved from terrestrial environments, which were predominantly from Actinobacteria [61, 72].

Experiments using this initial PCR primer set to retrieve *isoA* gene sequences from environmental samples and isoprene enrichments, together with new data from DNA stable isotope probing experiments (see below), indicated the considerable diversity of isoprene degraders in the environment. Further isolations of new isoprene degraders (Table 1) and analysis of their *iso* genes enabled redesign and refinement of *isoA* primers [73]. Carrión *et al*. [73] aligned *isoA* genes from 38 *bona fide* isoprene degraders available from GenBank, and *isoA* from our newly isolated isoprene degraders, including Actinobacteria (e.g. *Rhodococcus *, *Gordonia* and *Mycobacterium *), Alphaproteobacteria (e.g. *Sphingopyxis *) and Betaproteobacteria (e.g. *Variovorax *), together with 18 *isoA* sequences found in metagenomes retrieved from willow soil, willow leaves and poplar leaves. These newly designed *isoA*-specific PCR primers amplified *isoA* from DNA extracted from all positive-control isoprene degraders tested. They did not yield PCR amplicons with DNA from isoprene non-degraders that contained related SDIMOs such as soluble methane monooxygenase, toluene monooxygenase or alkene monooxygenase. The primers were also negative with DNA from isoprene non-degrading strains of *Rhodococcus* and *Variovorax,* thus confirming their specificity for *isoA* alone [73]. This primer set was then used to investigate the relative diversity of *isoA* sequences in a range of environmental samples, including leaves of isoprene-producing trees and adjacent soils, freshwater and coastal sediments and rubber-contaminated soil, to reveal the variation in diversity of isoprene degraders in these environments. Major genera represented included *Rhodococcus *, *Mycobacterium *, *Nocardioides *, *Gordonia *, *Sphingopyxis* and *Variovorax *, but a number of *isoA* sequences, while having a high degree of identity with *isoA* from extant isoprene-degraders, clearly pointed to as-yet uncultivated isoprene degraders being present in many of these environments. A quantitative PCR assay was also developed and tested to quantify *isoA* genes, normalized to 16S rRNA gene abundance, in the same environmental samples. Interestingly, soil from the vicinity of oil-palm and willow trees harboured the highest abundance of *isoA* genes, in the range of ~75–300 copies per million 16S rRNA gene copies [73]. These molecular tools are now being used to investigate the distribution, diversity and abundance of isoprene degraders in a wider range of environmental samples and provide powerful assays for targeted enrichment strategies to isolate new isoprene degraders.

Another powerful cultivation-independent tool for identifying active isoprene degraders in samples from the environment is DNA-SIP. This relies on incubation of environmental samples with ^13^C-labelled isoprene, isolating the resulting heavy ^13^C-labelled DNA from microbes in the sample that grow on isoprene, and then analysing this heavy DNA using a range of techniques (reviewed in Dumont and Murrell [74] and Dumont and Hernandez Garcia [75]). The first DNA-SIP experiments with ^13^C-isoprene in the terrestrial environment were carried out by El Khawand *et al*. [72], who examined soil from the vicinity of willow (*Salix fragilis*) trees. PCR assays, with the original set of primers designed for *isoA* sequences, on ^13^C-labelled DNA arising from SIP experiments indicated a variety of actinobacterial isoprene degraders were present. However, 16S rRNA-based assays with ^13^C-DNA hinted that there was considerably more diversity of isoprene degraders in these soils, with sequences from members of the Betaproteobacteria, including *Comamonas* and *Variovorax *, being detected. Similar DNA-SIP experiments with surface sediment samples from the Colne Estuary also revealed an abundance of actinobacterial isoprene degraders such as *Mycobacterium *, *Gordonia* and *Rhodococcus *, but also hinted at the possibility that Gram-negative isoprene degraders might be present [61].

Focussed metagenomics experiments, combining ^13^C-isoprene DNA-SIP with metagenomics and analysis of ^13^C-labelled DNA, were first carried out with phyllosphere samples [50]. Leaf washings of the high isoprene-emitting white poplar (*Populus alba*) were incubated in microcosms with ^13^C-labelled isoprene. Microcosms rapidly assimilated the isoprene, and after CsCl gradient centrifugation, the heavy DNA arising from the SIP experiments was used in shotgun metagenomics experiments. The ^13^C-labelled community was dominated by *Rhodococcus* species together with Proteobacteria of the genus *Variovorax *. Genome reconstitution through binning of ^13^C-DNA sequences and examination of these genomes for *iso* genes enabled the capture of near complete genomes of isoprene-degrading *Rhodococcus* species and also the genome of a new putative isoprene-degrading *Variovorax* species, which contained the metabolic gene clusters *isoABCDEF* and *isoGHIJ*. In order to prove that the *iso* genes *isoABCDEF* from this *Variovorax* strain encoded a *bona-fide* IsoMO, they were expressed in a *Rhodococcus* AD45 mutant, which had been cured of the 300 Mbp plasmid enabling isoprene metabolism and which could not oxidize isoprene. Expression of *isoABCDEF* in this mutant conferred the ability to oxidize isoprene, thus proving that these genes were indeed functional [50]. Furthermore, metatranscriptome analysis with RNA isolated from the same DNA-SIP microcosms that were actively degrading isoprene confirmed that these *Variovorax iso* genes were expressed under the enrichment conditions. These molecular biology data informed further targeted enrichments, which resulted in the isolation of isoprene-degrading *Variovorax* strains from soil and leaves [50–52], and have provided the Gram-negative ‘workhorse’ strain *Variovorax* WS11, which is currently being characterized at the physiological and biochemical level, thus complementing our metabolic studies on isoprene metabolism in *Rhodococcus* AD45 [76]. Similarly, DNA-SIP experiments with soil from the vicinity of willow trees, also known to be high emitters of isoprene, and subsequent metagenomics analysis of ^13^C-labelled DNA, revealed an active and diverse population of isoprene degraders from this environment. This isoprene-degrading community contained *Rhodococcus,* but interestingly was dominated by proteobacterial isoprene-degraders. Targeted enrichments have again resulted in the isolation of further novel isoprene-degrading bacteria including strains of the genera *Ramlibacter *, *Sphingopyxis* and *Nocardioides* as well as previously isolated *Rhodococcus* strains, thus adding to the growing database of isoprene-degrading bacteria, their genomes and *iso* genes [52].

## Perspectives

A significant unanswered question is the extent to which microbes in the environment contribute to the global isoprene cycle. Cultivation-independent methods are yielding valuable information on the distribution, diversity and activity of isoprene-degraders in environmental samples and quantitative methods are now available to assess abundance. What is now required is a systematic survey of the occurrence and types of isoprene-degrading bacteria in contrasting environments, for example, the phyllosphere and soil in the vicinity of high isoprene-emitting trees, versus non-isoprene emitters. Of particular interest will be ‘hot-spots’ for isoprene production such as oil-palm plantations, where the tree canopy and surface soils will be rich in isoprene with respect to the atmosphere (and indeed of potential concern with respect to changes in air quality). Measurement of the *in situ* affinities for isoprene of microbes in these environments, together with isoprene flux measurements, will also be important to inform global models of the isoprene cycle. While it is clear that microbes have different capacities to degrade isoprene depending on its concentration (e.g. Singh *et al*. [54]), the original experiments of Cleveland and Yavitt [46], which revealed the capacity for soils to take up very low concentrations, need to be built on to establish if there are both ‘high affinity’ and ‘low affinity’ isoprene degraders in the environment, as was originally observed with aerobic methanotrophs [77].

Other environments that could prove fruitful for the study of isoprene degraders are those which may be influenced by global warming, such as Arctic environments, where thawing is predicted to increase isoprene emissions [36] and where warming should increase emissions from lakes [38]. Non-vascular plants such as mosses, which can emit isoprene [78], may also be an interesting environment to explore for isoprene degraders, as will be marine and freshwater environments, where algae may have been overlooked as a significant source of isoprene [21, 25, 38, 79].

A substantial collection of new isoprene degraders is available for laboratory studies to further examine the biochemistry of IsoMO, affinities, substrate specificities and the effects of various inhibitors on this novel enzyme. In addition, where *isoA* genes and other genes associated with the classical isoprene-degradation pathway cannot be detected, alternative mechanisms of isoprene degradation should be sought [80, 81]. The pathways of isoprene degradation are being studied in both *Rhodococcus* AD45 and *Variovorax* WS11, for which we have established protocols for genetic manipulation including mutagenesis, expression and reporter gene construction [50, 68, 76] (Dawson, Carrion *et al*., in preparation). It will be interesting to learn more about the mechanisms of regulation of isoprene oxidation in these strains and then translate this information back into the environment by carrying out *in vivo* studies investigating expression of isoprene oxidation pathways in the soil and phyllosphere. Identification of promoters and regulatory proteins controlling *iso* gene expression will also be invaluable for these studies in order to examine the contribution that isoprene degraders make to the global biological sink for isoprene. For example, in high isoprene environments, such as the phyllosphere of high-isoprene-emitting trees, do isoprene degraders remove isoprene at the surface of their leaves (or indeed as endophytes inside the leaves) before it is released to the atmosphere? The use of isoprene *gfp*-reporter strains that we have constructed (Carrion *et al*., unpublished) will assist in answering these questions, as will the use of single-cell genomics techniques coupled with Raman microspectroscopy, fluorescence *in situ* hybridization and SIP techniques [82].

A detailed characterization of the novel enzyme IsoMO and its comparison with other enzymes of the SDIMO family will also yield novel insights into its structure and catalytic mechanisms. Our preliminary analyses with IsoMO from *Rhodococcus* AD45 and *Variovorax* WS11 indicates that the substrate specificity of this enzyme for dienes and other alkenes is relatively broad [76] (Sims, Dawson *et al*., unpublished) and IsoMO may have potential as a biocatalyst for production of epoxides. In an environmental context, knowledge of specific inhibitors for IsoMO and isoprene oxidation will be important since other SDIMO enzymes, such as the soluble methane monooxygenase (sMMO) from methanotrophs, can co-oxidize isoprene, and thus there is a possibility that sMMO could contribute to the global isoprene sink, particularly in the terrestrial environment. Initial experiments with IsoMO suggest that acetylene, a potent inhibitor of methane monooxygenases [83], is not an effective inhibitor of isoprene oxidation by IsoMO [76], and so this may be used to eliminate any contribution of methanotrophs to isoprene oxidation by bacteria in environmental samples and thus enable estimates of the global sinks of isoprene, at least in soil environments. Comparisons of IsoMO and other related SDIMOs at the gene/protein sequence level will also be important to determine the evolution of IsoMO in relation to other SDIMOs and to investigate the possibility of horizontal gene transfer of *iso* genes in the environment, which could explain why isoprene oxidation appears to be a widespread trait in bacteria. Although isoprene oxidation does not appear to be found in Archaea or Eukarya, again this needs to be examined further using both cultivation-dependent and cultivation-independent techniques.
